# Training Primary Healthcare Professionals for Expanded Newborn Screening with Tandem Mass Spectrometry: Challenges for Community Genetics in Brazil

**DOI:** 10.3390/ijns11030051

**Published:** 2025-06-30

**Authors:** Luzivan Costa Reis, Tassia Tonon, Marina Bernardes Acosta, Simone Martins de Castro, Vivian de Lima Spode Coutinho, Débora Gusmão Melo, Ida Vanessa Doederlein Schwartz

**Affiliations:** 1 Postgraduate Program in Medicine: Medical Sciences, Faculty of Medicine, Universidade Federal do Rio Grande do Sul, Porto Alegre 90035-003, RS, Brazil; tassiatonon@gmail.com; 2 Faculty of Pharmaceutical Sciences, Universidade Federal do Rio Grande do Sul, Porto Alegre 90610-000, RS, Brazil; marinabacosta@hotmail.com (M.B.A.); simone.castro@ufrgs.br (S.M.d.C.); 3 Reference Service on Newborn Screening, Hospital Materno Infantil Presidente Vargas, Porto Alegre 90035-076, RS, Brazil; vivian.coutinho@portoalegre.rs.gov.br; 4 Genetics Division, Department of Morphology and Genetics, Escola Paulista de Medicina, Universidade Federal de São Paulo (UNIFESP), São Paulo 04023-062, SP, Brazil; dgmelo@unifesp.br; 5 Postgraduate Program in Genetics and Molecular Biology, Universidade Federal do Rio Grande do Sul, Porto Alegre 91501-970, RS, Brazil; 6 Medical Genetics Service, Hospital de Clínicas de Porto Alegre, Porto Alegre 90035-903, RS, Brazil; 7 Brazilian National Institute on Rare Diseases, InRaras, Porto Alegre 90000-000, RS, Brazil

**Keywords:** newborn screening, primary healthcare professionals, inborn errors of metabolism, basic health units

## Abstract

In Brazil, dried blood spots (DBSs) for newborn screening (NBS) should be collected between the 3rd and 5th days of life at local Basic Health Units (BHUs). This study reports the experience of face-to-face training at BHUs in southern Brazil during a pilot study for tandem mass spectrometry (MS/MS) inclusion in the NBS program. The pilot project involved screening for 22 inborn errors of metabolism (IEMs). The professionals at the BHUs were instructed to carry out the following: (a) explain the study to parents or guardians; (b) collect additional DBS samples on a different collection card (research card); and (c) deliver results to families. In-person visits were conducted at all 137 BHUs. These visits included an overview of the pilot project and distribution of educational materials, including a list of the 22 IEMs and informational leaflets on MS/MS-based NBS. Among the 486 healthcare professionals who participated, 91.2% were women. Overall, 97.1% of the BHUs reported being satisfied with the project. Questions regarding IEMs were raised in 40.1% of BHUs, and 13.1% reported complaints about the research card due to its lighter texture and drying difficulty. Training primary healthcare professionals in IEMs remains an urgent priority in Brazil, particularly in the context of expanded NBS using MS/MS, since they are the frontline professionals in the NBS program.

## 1. Introduction

Newborn screening (NBS) was first introduced in Brazil in 1976, initially for the detection of phenylketonuria (PKU). In 2001, the Brazilian Newborn Screening Program (BNBSP) was implemented as part of the Brazilian Unified Health System (in Portuguese: Sistema Único de Saúde—SUS) [1,2]. Coordinated by the Brazilian Ministry of Health, the BNBSP currently covers approximately 82.5% of Brazilian newborns [3,4]. Despite over two decades of implementation, significant regional disparities persist. The highest coverage rates are found in the southeast and south regions of Brazil [5].

NBS for inborn errors of metabolism (IEMs) using dried blood spots (DBSs) analyzed by tandem mass spectrometry (MS/MS) can simultaneously assess more than 40 metabolites and identify over 50 types of IEMs [6,7,8,9]. Currently, the BNBSP includes screening for seven diseases not analyzed by MS/MS: congenital hypothyroidism, PKU, sickle cell disease, cystic fibrosis, congenital adrenal hyperplasia, congenital toxoplasmosis, and biotinidase deficiency [10].

In 2021, Federal Law No. 14,154 was published, recommending the expansion of the screening panel to include MS/MS and genetic tests within the BNBSP. Although this expansion has not yet been implemented, it will gradually occur in four phases: (1) inclusion of galactosemias, aminoacidopathies, urea cycle disorders, and organic acidurias/fatty acid beta-oxidation disorders (FAODs); (2) inclusion of lysosomal disorders; (3) inclusion of primary immunodeficiencies; and (4) inclusion of spinal muscular atrophy [3,4,11]. NBS for aminoacidopathies, urea cycle disorders, and organic acidurias/FAODs depends on MS/MS [4].

The Brazilian Ministry of Health recommends that blood collection for the NBS should be performed between the 3rd and 5th day of the newborn’s life at local Basic Health Units (BHUs) [2]. The NBS is not considered mandatory in Brazil, but it is considered a national public health policy, such as the vaccination program. So, a formal consent process is not requested [1]. According to the BNBSP, “it is the responsibility of the prenatal care to inform and guide both the population and pregnant women on how and where to perform the NBS, emphasizing that this test should be conducted by the 5th day of the baby’s life. The nursing team in maternity wards, birthing houses, Indigenous Health Houses, and Multidisciplinary Indigenous Health Teams are expected to alert and guide the postpartum woman and her family about the need to perform the NBS test at the BHUs closest to their residence when the collection is not carried out there. If there is a refusal by family members to perform the test, the person in charge at the collection site should advise them about the risks of not conducting the examination. This fact should be documented with the signatures of the parents or guardians [1]”. At these BHUs, primary healthcare professionals are also responsible for referring families of newborns with positive NBS results for confirmatory tests and evaluations performed at a NBS Reference Center (usually, there is one NBS Reference Center in each Brazilian state). BHUs professionals must be adequately informed about NBS to effectively engage with families and ensure the continuity of care for the child [12,13]. A Brazilian study showed that BHUs professionals had an insufficient understanding of the BNBSP [13].

Despite the increasing global importance of NBS, particularly in low- and middle-income countries, the effective implementation of expanded NBS requires an integrative community genetics approach—one that considers the local social, educational, and infrastructural realities of primary healthcare settings. Therefore, the objective of this study was to report on the experience of conducting face-to-face training sessions for primary healthcare professionals during the implementation of a pilot project for expanded NBS in the city of Porto Alegre, state of Rio Grande do Sul (RS), southern Brazil.

## 2. Materials and Methods

### 2.1. Study Design

This is a descriptive study on the training carried out at BHUs in the municipality of Porto Alegre, RS, Brazil, between August 2021 and March 2022, as part of a pilot research study for the expansion of NBS in that municipality. This study was designed by a multidisciplinary team, which included health professionals from the local NBS and Rare Diseases Reference Centers, and was approved by the Institutional Review Boards (IRBs) of the Hospital de Clínicas de Porto Alegre (HCPA; CAEE 38921520.6.0000.5327; GPPG 2020-0571) and the Hospital Materno-Infantil Presidente Vargas (HMIPV). The IRBs waived the application of the consent form but requested the dissent form for this project. The project was intensively publicized in the local media throughout its implementation to make it known to the majority of the population.

### 2.2. Basic Health Units in Porto Alegre

BHUs are the “gateway” to the health system for the population served by the SUS. Previously known as Health Posts, Health Centers, and/or Family Clinics, these units are part of Primary Health Care (PHC) and are staffed by at least one physician, nurse, dentist, dental hygienist, nursing technicians, and community health agents. The functions of BHUs include providing essential services, such as consultations, exams, vaccinations, and procedures like blood collection, for NBS, which is mainly performed by nurses and nursing technicians (https://www.saude.df.gov.br/unidades-basicas, accessed on 11 June 2025).

**Figure 1:**
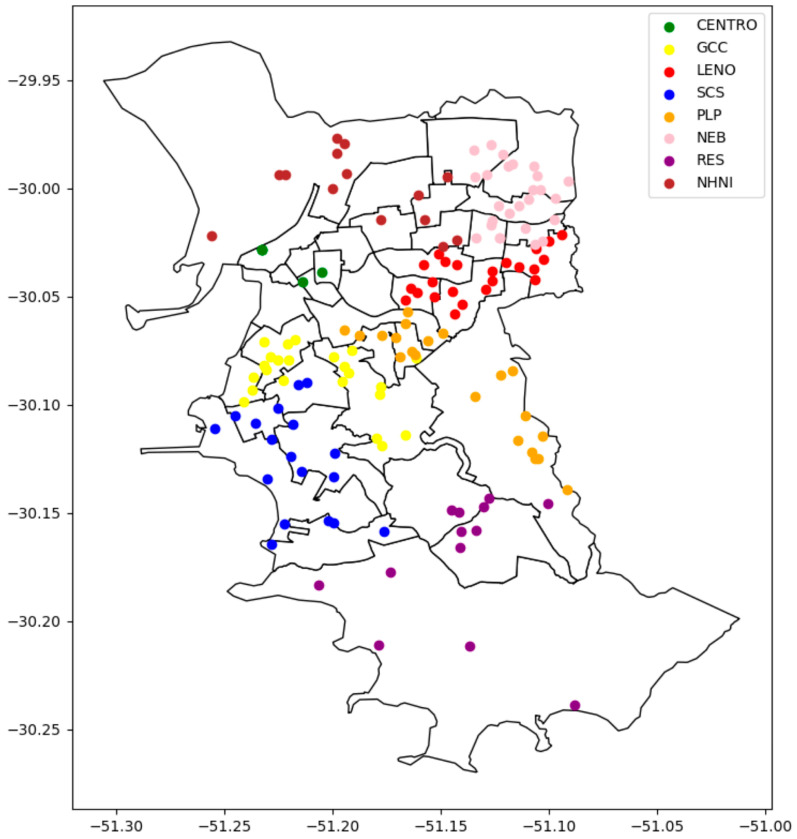
Geographic distribution of the 137 Basic Health Units in Porto Alegre, RS, Brazil, grouped by the eight District Administration regions. This spatial organization supported the planning of visits and implementation of MS/MS-based NBS during the pilot study. Note: The 137 Basic Health Units are linked to eight District Administrations (DAs)—CENTRO (Center, *n* = 5); (2) RES (Restinga/Extreme South, *n* = 15); GCC (Glória/Cruzeiro/Cristal, *n* = 20); PLP (Partenon/Lomba do Pinheiro, *n* = 14); NHNI (Northwest/Humaitá-Navegantes/Ilhas, *n* = 13); SCS (South/Central South, *n* = 18); LENO (East/Northeast regions, *n* = 21); and NEB (North/Eixo-Baltazar, *n* = 25).

Porto Alegre has an area of 495 km^2^ and a population of about 1,332,845 inhabitants [14]. It has 137 BHUs distributed throughout the city, and they are managed by the eight District Administrations (DAs): (1) CENTRO (Center, *n* = 5); (2) RES (Restinga/Extreme South, *n* = 15); (3) GCC (Glória/Cruzeiro/Cristal, *n* = 20); (4) PLP (Partenon/Lomba do Pinheiro, *n* = 14); (5) NHNI (Northwest/Humaitá-Navegantes/Ilhas, *n* = 13); (6) SCS (South/Central South, *n* = 18); (7) LENO (East/Northeast regions, *n* = 21); and (8) NEB (North/Eixo-Baltazar, *n* = 25). DAs are regional administrative structures that implement healthcare strategies at the local level through SUS. Figure 1 shows an updated plot map with the spatial distributions of BHUs by city neighborhoods, illustrating how BHUs are organized across the eight administrative regions.

### 2.3. The Pilot Study on Including IEM Screening by MS/MS in NBS in Porto Alegre

The pilot study began in June 2021 during the COVID-19 pandemic, and samples were collected from all newborns whose parents or guardians attended BHUs for this purpose. As part of the project, BHUs professionals, after collecting the DBS for the BNBSP, were expected to carry out the following: (a) explain the research to the parents; (b) collect additional DBS samples on a different collection card (research card); and (c) deliver the results report to the families.

Before the project started, all BHUs received training through an online meeting attended by at least one representative from each DA. The online training was conducted by the research team, led by both the director of the local NBS center (SMC) and the principal investigator (IVDS), via Google Meet. In the meeting, the objectives and methodology of the pilot study were first explained in detail. Afterwards, an open discussion was conducted to clarify any questions about the procedures presented. At the end, it was requested that each representative present at the meeting disseminate the information received, and e-mail addresses/phone numbers were provided for obtaining information and for clarifying further questions.

Additionally, the following materials produced by the research team were sent in advance to all BHUs: (a) a poster listing the full names of the 22 IEMs included in the pilot project [fatty acid metabolism and ketone bodies (*n* = 8), organic acidemias (*n* = 9), aminoacidopathies (*n* = 3), and urea cycle disorders (*n* = 2)]; (b) leaflets explaining the project and the NBS based on MS/MS; and (c) collection cards for the research (described in detail in Table 1)—the research card had 8 circles for blood collection, 2 more than the usual card, so 6 circles were for the analysis of the diseases covered by the BNBSP and 2 were for MS/MS (research). In addition to these materials, a refusal form was also provided in case parents chose not to authorize their newborn’s participation in the research.

After the project’s launch, a member of the research team conducted at least one in-person visit to each BHUs. The details of these visits are provided below.

### 2.4. Visits to BHUs

The managers of each health unit were contacted via email, telephone, and WhatsApp to schedule visits. There was no predefined minimum or maximum number of participants per visit. Conversations were held only with healthcare professionals available at the scheduled date and time and were not recorded. The mean number of participants per visit was 3.1 ± 1.0. Since the visits occurred during the pandemic period, there was a time restriction for their completion. The main objective was to have feedback on the execution of the project and clarify questions.

The first author (L.C.R.) of this study visited all BHUs and was welcomed into a designated room, where he initiated informal discussions with present healthcare professionals. Other research team members (T.T., M.B.A., S.M.d.C., V.d.L.S.C., and I.V.D.S.) participated in at least one visit. A brief presentation was given to attendees, covering the pilot study, IEMs, and NBS, and lasting between 10 and 45 min; this was also dependent on whether there were interruptions for answering questions. At the end of the discussion, participants were asked open-ended questions, including the following:

- “Have you experienced any challenges during the pilot study?”;
- “Have you encountered issues using the research collection card?”;
- “Do you know what an IEMs is?”;
- “Have you heard about NBS using MS/MS?”;
- “Where are the NBS materials that were previously sent?”;
- “What is your level of satisfaction with the implementation of the pilot study?”

For the last question, the answers could be ① Bad, ② Regular, ③ Intermediate, ④ Good, or ⑤ Excellent. All questions were asked orally, and the answers were noted by the researchers.

The initial medical reports of the research were not previously assessed regarding usability. They had 3 pages, containing a table with the name of all metabolites analyzed, the values found for each one, and the reference values. As a conclusion, the reports informed whether the test was normal or abnormal, without informing the disease for which the screening was positive. Because of the questions raised by the BHUs professional (Results Section), the reports were changed to a shorter 1-page version, containing the name of the diseases screened, the result of the screening (normal or abnormal), and the name of the disease for which the screening was positive.

**Figure 2:**
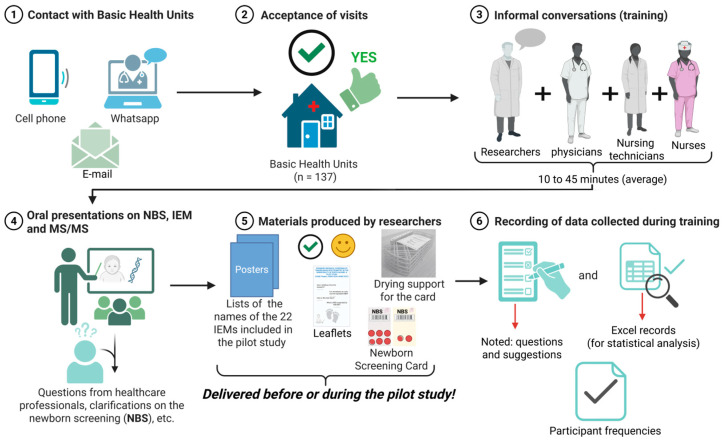
Step-by-step training strategy to prepare primary healthcare professionals for expanded newborn screening using tandem mass spectrometry (MS/MS) in Porto Alegre, RS, Brazil. Figure Created with BioRender.com.

A flowchart summarizing the educational materials, scheduling, visits, and feedback processes that structured the pilot study’s implementation is shown in Figure 2.

### 2.5. Statistical Analysis

Descriptive analyses were conducted using the Statistical Package for the Social Sciences (SPSS) version 18.0 (SPSS Inc., Chicago, IL, USA).

## 3. Results

Between August 2021 and March 2022, a total of 486 healthcare professionals participated in the training. Most of them were women (91.2%). Regarding the level of satisfaction with the implementation of the pilot study, in 97.1% (*n* = 133) of the BHUs, participants reported being satisfied (Table 2).

Data were collected during site visits and reflect subjective evaluations of training and project implementation. Complaints about the collection card (modified) used in the study were reported by 13.1% of the BHUs. The main complaint was that the collection card model was softer, had more circles (*n* = 8) for applying blood drops, and was more difficult to dry.

In 40.1% of the BHUs, healthcare professionals had questions about the IEMs included in the pilot study. The most frequent questions concerned the classification of these conditions, as well as the symptoms and treatments available. Regarding the educational materials sent in advance, 67.9% of the BHUs confirmed having received a poster and leaflet listing the names of the 22 IEMs screened. However, 32.1% reported not receiving these materials.

Other types of complaints were reported in 25.5% of the BHUs. In these cases, BHUs teams indicated difficulty in understanding the medical reports issued by the pilot study and noted that families often found these documents hard to interpret. They reported that the medical reports presented a lot of quantitative information, chemical data of the analytes analyzed in the MS/MS, and a very extensive table, without informing the name of the possible disease that the newborn could have in the case of a positive result. Additionally, concerns were raised about the financial burden (e.g., payment for transportation) faced by low-income families, especially when their newborns had positive results for IEMs—whether related to the pilot study or the regular BNBSP—and needed to go to a NBS Reference Center. In 10.2% of the BHUs, respondents suggested that the federal government should provide financial assistance to support these families in accessing follow-up care at the specialized centers.

Table 3 summarizes the perceptions and difficulties experienced during the pilot study in the BHUs.

## 4. Discussion

To our knowledge, this is the first study to explore in-person training to support the expansion of NBS using MS/MS at the primary healthcare level in Brazil. The predominance of female healthcare professionals observed in the BHUs aligns with findings from other Brazilian studies [13,15,16], reflecting broader gender patterns in primary healthcare and NBS-related services.

NBS is a global public health initiative aimed at improving neonatal outcomes and enhancing family well-being. To ensure its effectiveness, especially when expanding screening panels to include IEMs, healthcare professionals involved in sample collection must be adequately informed about these conditions and be able to educate families on the importance of early diagnosis and treatment. It is also essential that families receive clear and accessible information about the benefits of NBS and understand why it is important to return to the BHUs with their child, either for follow-up testing in the case of a positive initial result or simply to receive a normal result [17,18].

We observed that many professionals at the BHUs raised questions regarding the clinical presentation, genetic classification, prevalence, and treatment of IEMs. These inquiries reveal a critical educational gap, consistent with findings from a study in Hong Kong, where nearly half of the 210 surveyed healthcare professionals (50.0% nurses, 32.9% physicians, and 17.1% other professionals) reported not knowing which IEMs were included in the screening panel [19]. These findings mirror those from similar initiatives in other middle-income countries such as Pakistan and China, where healthcare providers also expressed limited knowledge about metabolic disorders included in NBS [9,20]. In this context, our findings suggest that BHUs professionals in Brazil also require targeted training to support the successful integration of IEMs into the BNBSP.

This knowledge gap aligns with previous studies that have also highlighted limitations in professional training for NBS. For instance, Mesquita et al. [13] found that over 70% of healthcare professionals in Uberaba, Brazil, had not received continuing education on this topic. Similarly, Newcomb et al. [21] reported that professionals in Texas lacked sufficient knowledge of NBS protocols and the proper use of collection cards. These findings reinforce the necessity of ongoing professional development. As Costa et al. noted [15], continuous education not only builds technical competencies but also strengthens communication between professionals and the communities served by BHUs. This aligns with the broader concept of lifelong learning in healthcare, which emphasizes that professional development should be a continuous, needs-driven process embedded in daily practice [22]. In this context, continuing education is not an isolated event but rather part of a permanent education strategy that fosters critical thinking, problem-solving, and interdisciplinary collaboration [23].

Nurses play a particularly central role in this context. Studies in Brazil and Jordan have shown that they are often the main sources of information for families regarding NBS [18,24], whether through direct explanations or by distributing informational leaflets during prenatal care [25]. Kasem et al. emphasized that this educational role must be continuously supported to foster greater family engagement [18]. To fulfill this role effectively, professionals must be equipped with scientific knowledge and communication skills that allow them to translate complex medical concepts into culturally and linguistically appropriate language for families. Educational programs should therefore include content on health literacy, communication techniques, and socioemotional competencies that enable professionals to support families in moments of vulnerability. Moreover, effective collaboration between clinical geneticists, pediatricians, and primary healthcare teams is essential for improving diagnostic accuracy and ensuring proper follow-up for patients with IEMs [26,27].

In our study, practical improvements were observed following the training visits. Although not quantified in this manuscript, we observed the improved completion of sample forms and a greater understanding of IEMs after visits to the BHUs. These outcomes align with findings by Amaral et al., who observed significant improvements in form completion rates after carrying out training in Goiás, Brazil [28]. In another study, Pedrini et al. found that community health agents in Rio Grande do Sul, Brazil [29], demonstrated measurable knowledge gains after receiving training on genetic disorders, with a focus on mucopolysaccharidoses. These examples reinforce the value of educational initiatives.

Nevertheless, some challenges were also identified. For instance, healthcare professionals reported difficulties in understanding the medical reports used during the pilot study, which limited their ability to communicate results to families. In response, we simplified the report’s language to improve comprehension. This finding is important because it reflects the importance of including primary healthcare professionals in the planning of research on NBS and of conducting literacy studies prior to the implementation of medical reports or educational materials. Unfortunately, we were not able to obtain the familial perspective on the quality of the medical reports. These challenges also emphasize the need for both technical training and educational support that equips professionals with tools to explain complex genetic conditions in lay language and adapt communication according to family profiles and health literacy levels. Additionally, logistical issues were reported, such as the lack of an appropriate structure for drying the softer filter paper introduced during the pilot. To address this, we developed and distributed 3D-printed drying supports to all BHUs in the municipality.

Another recurring concern involved the financial burden related to the costs of transportation faced by families who received positive NBS results and who needed to go to referral centers. This issue has been documented in other Brazilian regions. For example, Araújo et al. reported that transportation costs could represent nearly 20% of Brazil’s minimum wage [30]. Geographic barriers also intensify this problem: In the Amazon region, some families travel over 1000 km (up to 20 h) to access specialized care [31]. In the northeastern semiarid region, over half of low-income patients live more than five kilometers from a BHUs [32]. These findings support our observation that without policies to mitigate financial and geographic barriers, timely follow-up after a positive NBS result may be compromised, with serious implications for the health of affected infants.

Taken together, our findings have practical implications for optimizing the delivery of expanded NBS services at the primary healthcare level in Porto Alegre and potentially in other Brazilian settings. Promoting a culture of interprofessional education is essential to ensure that all members of the healthcare team—from nurses to community health workers—share a common understanding of the objectives, procedures, and challenges of expanded NBS. This collaborative approach has the potential to improve care coordination and patient outcomes, as highlighted by the World Health Organization’s recommendations for integrated service delivery in primary care settings [33]. Addressing training gaps through well-structured educational programs, as advocated by Mendes et al. [34], Tariq et al. [20], and Kasem et al. [18], can improve both professional practice and family acceptance of expanded NBS. Educational strategies should value the lived experiences of professionals and their local realities, promoting active learning approaches such as case discussions, role-playing, and team-based simulations. These findings suggest that the model implemented in Porto Alegre could serve as a template for similar initiatives in other Brazilian municipalities, particularly those with limited access to specialized genetic services. It is not known how many couples choose not to perform the NBS on their children in the country, nor whether the couples who opt for it truly understand the test and its implications. In any case, it is expected that the more informed the BHUs professionals are on the subject, the greater the likelihood that couples will make a truly informed decision.

Currently, several strategies are available in Brazil to support education in medical genetics and NBS. The Brazilian Society of Medical Genetics and Genomics (SBGM) has published two educational booklets aimed at primary healthcare professionals and offers specific training courses during its annual congresses [35,36]. Similarly, the Brazilian Society of Newborn Screening (SBTEIM) provides a user-friendly disease dictionary that explains the seven conditions already included in most state-level screening programs [37]. These resources are written in accessible language, serving as valuable tools for both healthcare providers and families and thereby supporting broader health promotion efforts. We believe that the inclusion of MS/MS in NBS in Brazil should be accompanied by the development of investigation and monitoring protocols for each disease with a positive screening result. These protocols should be simplified, disease-specific, and easily accessible on the Ministry of Health’s website to support healthcare professionals during result disclosure and follow-up. Real-time support through telemedicine services connected to BHUs could further assist professionals in interpreting medical reports related to NBS, IEMs, MS/MS, and other conditions [38].

The findings of this pilot study underscore the urgent need for national policies that prioritize continuous education of primary healthcare professionals in NBS, particularly in preparation for the phased expansion of the Brazilian NBS program. Federal and state health authorities should allocate dedicated funding for training programs, the development of user-friendly educational materials, and logistical support to ensure consistent blood collection practices across regions. Additionally, equity-focused policies are needed to reduce the financial and geographic barriers faced by families referred to specialized centers. Together, these measures will be essential for scaling up MS/MS-based NBS and ensuring timely diagnosis and follow-up for affected newborns, particularly in underserved and remote communities.

### Limitations

This study has some limitations. First, professionals from BHUs did not participate in the design of the study. Second, although training was provided in multiple formats, we did not use structured tools, such as validated questionnaires or knowledge assessments, to measure the effectiveness of the training in terms of knowledge acquisition or behavioral change. Third, no prior usability testing of the medical reports was conducted before the pilot implementation, which led to comprehension issues among healthcare providers and families. These limitations highlight the need for co-design methodologies and systematic evaluation tools in future implementation research.

Finally, the COVID-19 pandemic was a significant constraint, as the healthcare professionals were on the front lines and primarily focused on vaccinating the population. Therefore, online training was conducted prior to the start of the pilot study, while in-person training was provided to BHUs management during the implementation of the pilot. Despite these challenges, the pilot provided valuable insights for the development of more inclusive and evidence-based training strategies for the expansion of NBS in Brazil.

## 5. Conclusions

This study demonstrates that face-to-face training plays a fundamental role in the implementation and optimization of expanded NBS, particularly in enhancing the understanding of IEMs within the public health system. Our findings highlight the need to simplify communication tools and engage healthcare workers more directly in the design of educational strategies. Based on our experience in Porto Alegre, in-person visits to BHUs contributed to professional development and improved the understanding of the expanded NBS process. We also suggest that continuing education initiatives for primary healthcare professionals should be further developed to enhance the support provided to mothers and newborns throughout the screening process.

To scale up NBS effectively, policy-level actions are urgently required. These include sustained federal funding, the standardization of educational protocols, and equitable resource allocation for BHUs across Brazil. Strategies such as telemedicine support and real-time consultation tools could enhance decision-making capacities among BHUs professionals when managing doubtful cases. Training public healthcare professionals in IEMs remains an urgent priority and represents a continuing challenge for health education in Brazil.

These findings highlight the need to integrate genetic screening into Brazil’s broader public health strategy, with strong institutional support to ensure long-term sustainability. The model implemented in Porto Alegre may serve as a replicable strategy for other municipalities in Brazil seeking to expand NBS in alignment with national public health goals.

## Figures and Tables

**Table 1:** Educational materials and research items produced and distributed during in-person training visits to 137 Basic Health Units (BHUs) in Porto Alegre, RS, Brazil (2021–2022), aimed at supporting healthcare teams in implementing expanded newborn screening using tandem mass spectrometry (MS/MS) during the pilot study.

Items Produced by the Pilot Study	Basic Description of the Materials Presented in the Training
Posters	One poster for each BHUs listing the full names of the 4 IEMs groups (metabolism of fatty acids and ketone bodies [*n* = 8 diseases]; organic acidemia [*n* = 9 diseases]; aminoacidopathies [*n* = 3 diseases]; disturbances in the urea cycle [*n* = 2 diseases]).
Leaflets	Details of expanded neonatal screening by MS/MS, research telephone contacts, email addresses, etc.
Collection cards (modified)	More malleable paper than the collection cards used by the municipality. Collection card (modified) for use in research contained two copies (yellow and white card, with 8 circles printed on the filter paper attached to the card), with a label and place to describe the neonatal collection

Note: Basic Health Units (BHUs), inborn errors of metabolism (IEMs), and tandem mass spectrometry (MS/MS).

**Table 2:** Satisfaction levels among healthcare professionals from Basic Health Units regarding the pilot study on expanded newborn screening using tandem mass spectrometry in Porto Alegre, RS, Brazil.

	N (137)	Frequency (%)
① Bad	0	0
② Regular	0	0
③ Intermediate	4	2.9%
④ Good	43	31.4%
⑤ Excellent	90	65.7%

**Table 3:** Summary of perceptions, challenges, and suggestions reported by healthcare professionals at 137 Basic Health Units in Porto Alegre, RS, Brazil, during the implementation of the pilot study on expanded newborn screening using tandem mass spectrometry.

Main Subjective Questions	Yes	No
Complaints about collection cards (modified) ^1^	18 (13.1%)	119 (86.9%)
Questions about IEMs ^2^	55 (40.1%)	82 (59.9%)
There was a poster or leaflet ^3^	93 (67.9%)	44 (32.1%)
Other complaints ^4^	35 (25.5%)	102 (74.5%)
Financial resources for families ^5^	14 (10.2%)	123 (89.8%)

Note: IEMs—inborn errors of metabolism; ^1^—main complaint: the collection card model was softer, contained more blood application circles (*n* = 8), and was more difficult to dry; ^2^—main questions: classification of IEMs, symptoms, and available treatments. ^3^—BHUs that did not receive the pilot study poster or leaflet; ^4^—difficulties from health professionals in understanding the medical reports issued by the research team, also reported by families; ^5^—suggestions that the federal government should support low-income families of newborns with positive NBS results in accessing specialized care.

## Data Availability

Data are unavailable due to privacy.
